# A Novel Approach for Discovering Condition-Specific Correlations of Gene Expressions within Biological Pathways by Using Cloud Computing Technology

**DOI:** 10.1155/2014/763237

**Published:** 2014-01-22

**Authors:** Tzu-Hao Chang, Shih-Lin Wu, Wei-Jen Wang, Jorng-Tzong Horng, Cheng-Wei Chang

**Affiliations:** ^1^Graduate Institute of Biomedical Informatics, College of Medical Science and Technology, Taipei Medical University, Taipei 110, Taiwan; ^2^Department of Computer Science and Information Engineering, College of Engineering, Chang Gung University, Taoyuan 333, Taiwan; ^3^Department of Computer Science and Information Engineering, National Central University, Taoyuan 320, Taiwan; ^4^Department of Biomedical Informatics, Asia University, Taichung 413, Taiwan; ^5^Department of Information Management, Hsing Wu University, New Taipei City 244, Taiwan

## Abstract

Microarrays are widely used to assess gene expressions. Most microarray studies focus primarily on identifying differential gene expressions between conditions (e.g., cancer versus normal cells), for discovering the major factors that cause diseases. Because previous studies have not identified the correlations of differential gene expression between conditions, crucial but abnormal regulations that cause diseases might have been disregarded. This paper proposes an approach for discovering the condition-specific correlations of gene expressions within biological pathways. Because analyzing gene expression correlations is time consuming, an Apache Hadoop cloud computing platform was implemented. Three microarray data sets of breast cancer were collected from the Gene Expression Omnibus, and pathway information from the Kyoto Encyclopedia of Genes and Genomes was applied for discovering meaningful biological correlations. The results showed that adopting the Hadoop platform considerably decreased the computation time. Several correlations of differential gene expressions were discovered between the relapse and nonrelapse breast cancer samples, and most of them were involved in cancer regulation and cancer-related pathways. The results showed that breast cancer recurrence might be highly associated with the abnormal regulations of these gene pairs, rather than with their individual expression levels. The proposed method was computationally efficient and reliable, and stable results were obtained when different data sets were used. The proposed method is effective in identifying meaningful biological regulation patterns between conditions.

## 1. Introduction

Using microarray technology combined with computational analysis is one of the most efficient and cost-effective methods for studying cancer. Using this method has enabled scientists to investigate and understand a vast array of cancer information [1–5], and it is used to analyze the functionality of specific genes during the development of a disease. In a high-throughput and parallel manner, expression profiling is performed by monitoring the expression levels for the thousands of genes that are simultaneously on an array. One of the most common and extensively used methods for determining the biological significance of genes when comparing cancerous and normal conditions is the identification of differential gene expressions. Multiple technologies, such as cloud computing, parallel systems, and data analysis strategies, have been developed to identify differential gene expressions by using microarray gene expression data [6–10].

In microarray experiments, the gene expression levels of the samples can be detected using the intensity of probes [6]. Heretofore, most studies have focused only on finding the differential gene expressions of various conditions; however, lack of methods focused on analyzing the correlations of differential gene expressions between conditions. Therefore, some abnormal regulations causing the diseases might have been disregarded. In addition, the major challenge accompanying this broad approach is computational complexity, because too many differential gene expressions (probes) must be calculated.

Cloud computing and parallel processing are considered valuable techniques because using them can greatly reduce the computation time of a program by efficiently combining multiple computers and processors in parallel. Therefore, we implemented cloud computing techniques to complete the program in considerably little time. Apache Hadoop is a distributed parallel data-processing framework that supports MapReduce-type computations, enabling users to perform distributed computations effectively in increasingly brittle environments [11]. We propose an approach for discovering the condition-specific correlations of gene expressions within biological pathways that implement an Apache Hadoop cloud computing platform. Three microarray data sets of breast cancer were collected from the Gene Expression Omnibus (GEO), and pathway information from the Kyoto Encyclopedia of Genes and Genomes (KEGG) was applied for discovering meaningful biological correlations.

## 2. Related Work 

Previously, several methods have been developed to identify differential gene expressions in biological pathways. Most of these methods focus on diagramming gene expression levels and calculating the correlation of clustered genes. Kanehisa et al. proposed an approach, Pathway Miner, which extracts gene-association networks from molecular pathways for predicting the biological significance of gene expression microarray data [12]. When pathways are extracted from Pathway Miner, the levels of gene expression can be discerned, but four samples could be shown at a time. ArrayXPath functions by focusing on mapping and visualizing microarray gene-expression data with biomedical ontologies and integrated biological pathway information and by displaying the results using Scalable Vector Graphics [13]. The genes can be annotated using different colors in a pathway according to condition. In addition, ArrayXPath can be used for conducting clustering analysis of the time series data of gene expression. Another function, PathMesh, facilitates analyzing the association between gene and disease terms by using MeSH. Thus far, however, lack of tool exists for clearly discerning coexpressional changes under different conditions.

The KEGG consists of a suite of databases that record an extensive amount of information on genes, enzymes, and regulation pathways [12], facilitating the retrieval of gene names and information on their interactions within biological pathways. The KEGG application programming interface (API) is essential for accessing the KEGG system and enables searching and computing the biochemical pathways involved in cellular processes or the analysis of all genes from a completely sequenced genome.

Apache Hadoop is an open source implementation of the Google MapReduce technology that simplifies programming tasks by automatically performing duties such as job scheduling, distributed aggregation, and fault tolerance [11, 13]. The Apache Hadoop software library is a framework that facilitates the distributed processing of enormous data sets across clusters of computers. Apache Hadoop involves using simple programming models such as the Hadoop Distributed File System, which provides high-throughput access to application data and duplicates the data on multiple nodes so that failures of nodes containing a portion of the data do not affect the computations [14]. Apache Hadoop is designed to scale from single servers to thousands of machines, with each offering local computation and storage. Recently, Hadoop platform has been widely applied for cloud computing of biological, genomics, and drug design [15–19].

## 3. Materials

Several experimental studies have examined the genetic profiles of breast cancer samples, and most of them have focused on identifying the differential gene expressions between relapse and nonrelapse breast cancer samples [20–24]. Previous studies have not identified the correlations of differential gene expressions between different conditions; therefore, some abnormal regulation correlations may not have been detected. The proposed approach focuses on discovering the condition-specific correlations of gene expressions within biological pathways, thus providing a more macroscopic result than that from using a single-gene approach and potentially facilitating the discovery of greater biological meaning from a microarray data. We collected three breast-cancer-related data sets (GSE2034, GSE1456, and GSE4922) from multiple arrays from the GEO [25] to determine the correlations of differential gene expressions between relapse and nonrelapse breast cancer samples. In addition, GSE2109, of which numerous samples were available, was used for examining the performance of the cloud computing platform.  Table 1 lists the information and materials extracted from the GEO.

## 4. Methods

**Figure 1:**
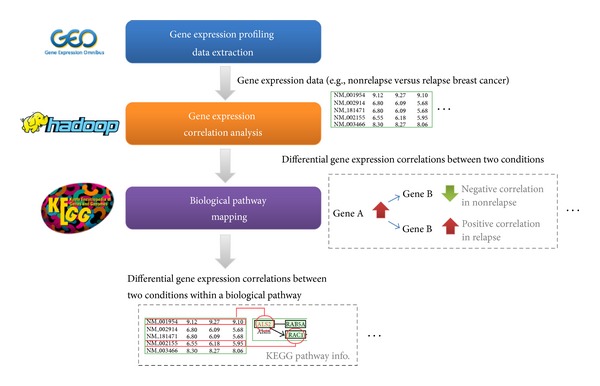
Overall flow of the proposed approach.

The system flow of the proposed approach is depicted in Figure 1. Three microarray data sets of breast cancer were collected from the GEO. An Apache Hadoop cloud computing platform was implemented for decreasing computation time, and pathway information from the KEGG was applied for discovering meaningful biological correlations. The aim of this study was to develop a system that can identify abnormal regulations within the biological pathways of the relapse and nonrelapse breast cancer samples by using microarray data. These selected differential correlations can facilitate identifying the factors involved in breast cancer relapse. The system was divided into three major parts: receiving and preprocessing data, analyzing gene expression correlations, and mapping condition-specific correlations within biological pathways.

The microarray data were extracted from the GEO and segregated into a nonrelapse condition and relapse condition according to the sample descriptions. Gene profiling was performed using Affymetrix U133A arrays, and microarray quality control was performed using an R package *affyQCReport* [26]. The *gcrma* function of the R package *affy* was applied to normalize the CEL files by using the Robust Multiarray Averaging (RMA) method [27].

The master node divides the computation into multiple smaller subproblems and distributes them to worker nodes. The master node collects all of the answers submitted by the worker nodes and combines these answers into the output result. Two MapReduce programs were implemented to compute the linear regression validation and correlations for each pair of genes based on Hadoop Version 1.1.1. We used our programs to conduct a performance evaluation on a Hadoop cluster of 10 Xen virtual machines, where nine are the data nodes and one is the name node. Based on our settings, every two virtual machines were deployed on a physical machine. A physical machine was equipped with two CPUs with Intel Xeon E5504 4C 2.0 GHz and 16 GB of RAM; each virtual machine for the data nodes was equipped with a virtual CPU (VCPU) with two cores and 1 GB of RAM; the virtual machine for the name node was equipped with a VCPU with four cores and 4 GB of RAM. To evaluate the scalability of our MapReduce implementations, we also implemented two corresponding sequential Java programs as the basis for performance comparisons. In the performance evaluation experiments, we partitioned the data set into several pieces, uploaded them to the Hadoop file system, and submitted several MapReduce jobs to analyze the pieces of data.

**Figure 2:**
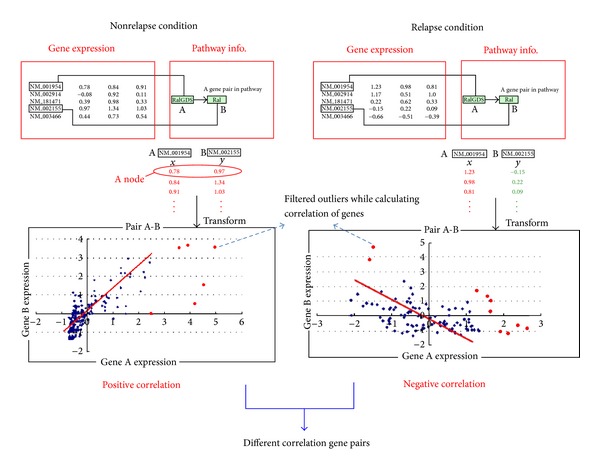
The process flow for identifying differential correlation of gene expression.

The KEGG [25] Java APIs were used to obtain both pathway and interaction data. A gene map may possibly have more than one accession number, and we used the KEGG API to map the gene designations according to the KEGG Markup Language (KGML) file of each pathway. After mapping the gene expressions and interacting gene pairs, we identified substantial differences under the conditions of nonrelapsed and relapsed. The detailed steps are shown in Figure 2.

For example, the correlation of the gene pair A-B was calculated under different conditions. First, we used the expression intensity of genes A and B as the values of the *x*-axis and *y*-axis, respectively, for drawing a node. The number of nodes represented the number of samples. Subsequently, linear regression analysis was applied to discard outliers based on the least square value of each node. If the least square value of a node is greater than the average least square value-added threefold standard deviation, then the node would be discarded when calculating the correlation of gene expression. Eighty percent of the data was reserved, at least for the setting of the threshold, to make the data more representative.

After discarding the outliers, Pearson's correlation coefficients were calculated for each gene pair. Pearson's correlation, *r*, ranges between −1 and 1, and the greater the value of *r*, the stronger the coexpression between the members of the gene pair. In this study, coexpressed genes were defined as genes with a correlation greater than 0.45, and reverse-expressed genes were defined as genes with a correlation less than −0.45. Unrelated genes were defined as genes with a correlation between −0.45 and 0.45. Genes with differential correlations between the nonrelapse and relapse conditions were collected when the difference of the correlation of the genes in the two conditions was greater than AVG + 3∗SD or less than AVG − 3∗SD, where AVG and SD are, respectively, the average and standard deviation of the correlations of differential gene expressions between the two conditions.

## 5. Results

### 5.1. Performance Evaluation of Cloud Computing

GSE2109 was used for evaluating the performance of the cloud computing platform. The size of the data set for analysis was 54,675 (probes) × 2,158 (samples) of floating numbers. Our MapReduce implementation for linear regression validation and correlation computation is a map-only program. It iteratively issues a MapReduce job by setting the number of Reducer to be zero, and the number of iterations depends on the input data size. That is, the outputs of the map tasks are written directly to the files system. At each iteration, our MapReduce implementation generates different number of map tasks for different input size. Based on our Hadoop MapReduce setting, it generates 2, 3, 6, 12, and 17 map tasks for 5,000, 10,000, 20,000, 40,000, and 54,675 probes, respectively.

**Figure 3:**
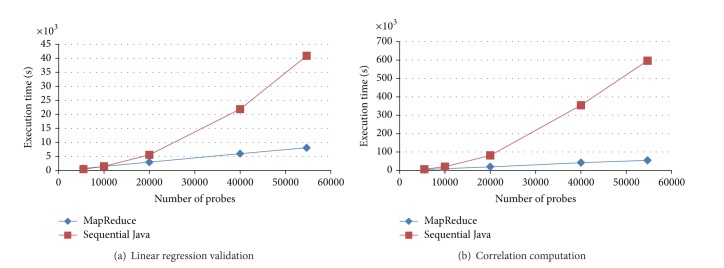
The execution times of linear regression validation and correlation computation by using the Hadoop MapReduce implementation and the sequential Java implementation, given different numbers of genes.

Figure 3(a) shows the execution times of linear regression validation for each pair of genes by using the Hadoop MapReduce implementation and the sequential Java implementation, given different numbers of genes. Based on the measured performance values, the MapReduce program was generally faster than the sequential Java program that was executed on the name node, particularly when the input data became extremely large. The sequential Java program was faster than the MapReduce program when the number of genes was smaller than 10,000. In addition, the execution time of the MapReduce program exhibited a linear growth for different numbers of genes, indicating that the Hadoop MapReduce framework was scalable in this case. By contrast, the execution time of the sequential Java program represented a quadric growth.  Figure 3(b) shows a similar result to those of Figure 3(a), indicating that the Hadoop MapReduce framework is scalable for computing the correlations of gene pairs. The only difference is that the correlation computation jobs are more CPU-intensive than the linear regression validation jobs. The MapReduce program can accomplish tasks approximately 10 times as fast as the sequential Java program can.

### 5.2. Analysis Results of Differential Gene Expression Correlations between Relapse and Nonrelapse Samples of Breast Cancer

The number of differential gene expression correlations between the relapse and nonrelapse breast cancer samples from GSE2034, GSE1456, and GSE4922 is shown in Table S1 (see the Supplementary Material available online at http://dx.doi.org/10.1155/2014/763237), and Table 2 lists the differential gene expression correlations in the pathways, and there were 33, 32, and 50 gene expression correlations mapped in the KEGG pathways of GSE2034, GSE1456, and GSE4922, respectively. The pathways of the differential gene expression correlations are listed in Table 3. Several known cancer-related pathways were identified, including pathways in cancer, the PI3K-Akt signaling pathway, MAPK signaling pathway, and Wnt signaling pathway. The results showed that the number of correlations decreased as the number of samples increased. For example, there were 40 and 107 relapse samples in GSE1456 and GSE2034, respectively, and there were 3,630,906 and 1,595,963 positive correlations discovered within GSE1456 and GSE2034, respectively. These results indicated that using more samples may conduct more reliable correlations within pathways. As shown in Table 3, two pathways, pathways in cancer and the PI3K-Akt signaling pathway, contained four correlated gene expressions between the relapse and nonrelapse breast cancer samples, and we used pathways in cancer for the demonstration and discussion of the discovery results presented in the following section.

### 5.3. Differential Gene Expression Correlations between Relapse and Nonrelapse Samples of Breast Cancer in Pathways in Cancer of the KEGG

As shown in Figure S1, four correlations of gene expression, NFKB2-PTGS2, JUN-MMP1, RUNX1-CEBPA, and JUN-FIGF, were identified in pathways in cancer. Table S2 shows the average log2-fold change in the gene expressions of NFKB2, PTGS2, JUN, MMP1, RUNX1, CEBPA, and FIGF between the relapse and nonrelapse samples, which were 0.02, −0.13, −0.16, 0.7, −0.08, −0.01, and −0.09, respectively. It shows that the differences of gene expression values between the two conditions were not substantial in these genes. However, the correlations between these genes were substantially different, and the differential correlations of NFKB2-PTGS2, JUN-MMP1, RUNX1-CEBPA, and JUN-FIGF were 0.37, 0.29, 0.29, and −0.27, respectively. The results imply that breast cancer recurrence may be induced by the abnormal regulations of these gene pairs, rather than their individual expression levels.

NF-kappa-B is a pleiotropic transcription factor and can be initiated by a vast array of stimuli related to many biological processes such as inflammation, immunity, differentiation, cell growth, tumorigenesis, and apoptosis through a series of signal transduction events. PTGS2 is regulated by specific stimulatory events and is responsible for the prostanoid biosynthesis involved in inflammation and mitogenesis. JUN is a putative transforming gene of avian sarcoma virus 17 and interacts directly with specific target DNA sequences to regulate gene expression. JUN is intronless and mapped to 1p32-p31, which is a chromosomal region involved in both translocations and deletions in human malignancies. The proteins of the matrix metalloproteinase (MMP) family are involved in the breakdown of the extracellular matrix in normal physiological processes, such as tissue remodeling, embryonic development, and disease processes, such as arthritis and metastasis. Core binding factor (CBF) is a heterodimeric transcription factor binding to the core element of many enhancers and promoters. Chromosomal translocations involving CBF are well documented and have been discovered to be associated with several types of leukemia. The protein encoded by FIGF is a member of the platelet-derived growth factor/vascular endothelial growth factor (PDGF/VEGF) family and is active in angiogenesis, lymphangiogenesis, and endothelial cell growth.

As mentioned, most of the discovered genes were related to cancer pathways and cancer regulations, including immunity, cell growth, tumorigenesis, and apoptosis. Although the gene expressions between relapse and nonrelapse samples were not substantially different, their correlations were substantially different. Thus, we believe these regulations of genes may be essential for regulating breast cancer recurrence.

## 6. Discussion and Conclusions

This study proposes an approach for discovering condition-specific correlations of gene expressions. An Apache Hadoop cloud computing platform was implemented to reduce the computation time. By using microarray data from the GEO, we discovered numerous differential gene expression correlations between the nonrelapse and relapse conditions of breast cancer. The results show that breast cancer recurrence is highly associated with the abnormal regulation of these gene pairs, rather than their individual expression levels. The pathways in cancer specifically show that NKFB2-PTGS2, JUN-FIGF, and RUNX1-CEBPA possess higher correlations of gene expression in nonrelapse samples and that JUN-MMP1 possesses higher correlations of gene expression in relapse samples. In addition, using the cloud computing technology successfully reduces the time required for conducting gene expression correlation analysis of microarray data, and it can be further applied for analyzing the correlation between different transcript isoforms using RNA sequencing data, which is helpful for deciphering the regulatory mechanisms of genes. The results show that our method is effective and can be extended to areas of biological analysis beyond that of breast cancer nonrelapse and relapse. We believe that the proposed method is effective for identifying meaningful biological regulation patterns between conditions and can be applied for developing coexpression networks and protein-protein interactions in the future.

## Supplementary Material

Figure S1. The differential correlation of gene expression between relapse and nonrelapse samples in pathways in cancer of the KEGG.
Table S1: Correlations of gene expressions between nonrelapse and relapse samples in three data sets.
Table S2: Gene expression correlations between relapse and nonrelapse samples in Pathways in Cancer.

## Figures and Tables

**Table 1:** Statistical data of used data set.

GEO no.	Sample no. (nonrelapse/relapse)	Platform	Probe no.	Description	Reference no.
GSE2034	288 (179/109)	Affymetrix U133a (GPL96)	22,283	Breast cancer	[20]
GSE1456	159 (119/40)	Affymetrix U133a (GPL96)	22,283	Breast cancer	[21]
GSE4922	249 (160/89)	Affymetrix U133a (GPL96)	22,283	Breast cancer	[22]
GSE2109	2,158	Affymetrix U133a Plus 2.0 (GPL570)	54,675	Different types of cancer	[23]

**Table 2:** Correlations of gene expressions between nonrelapse and relapse samples in three data sets mapped in the KEGG pathway.

	Condition (no. of samples)	Number of correlated gene pairs mapped in the KEGG pathway		Number of differential correlations of gene pairs (AVG ± 3∗SD)
		Positive (+) Cor. > 0.45	Negative (−) Cor. < −0.45	
GSE2034	Nonrelapse (179)	606	35	33
	relapse (107)	473	21	
GSE1456	Nonrelapse (119)	491	21	32
	relapse (40)	747	133	
GSE4922	Nonrelapse (160)	575	40	50
	relapse (89)	677	84	

**Table 3:** Pathways containing different correlated genes between relapse and nonrelapse breast cancer patients.

ID	Pathway names	Differential correlation genes (cor_Dif*)
hsa05200	Pathways in cancer	NFKB2 → PTGS2 (+0.37)
		JUN → MMP1 (+0.29)
		RUNX1→ CEBPA (+0.29)
		JUN → FIGF (−0.27)

hsa04151	PI3K-Akt signaling pathway	FGF4 → EGFR (−0.32)
		IRS1 → PIK3CB (−0.3)
		CSF1 → KIT (+0.3)
		EFNA1 → IGF1R (−0.28)

hsa04722	Neurotrophin signaling pathway	IRS1 → PIK3CG (+0.34)
		RPS6KA2 → NRAS (+0.3)
		IRS1 → PIK3CB (−0.3)

hsa04062	Chemokine signaling pathway	GNB5 → PIK3R5 (+0.31)
		CCL18 → XCR1 (−0.29)

hsa04150,	mTOR signaling pathway	
		
hsa04910,	Insulin signaling pathway	IRS1 → PIK3CG (+0.34)
		
hsa04930,	Type II diabetes mellitus	IRS1 → PIK3CB (−0.3)
		
hsa04960	Aldosterone-regulated sodium reabsorption	

hsa04010	MAPK signaling pathway	DUSP2 → MAPK8 (−0.34)

hsa04060	Cytokine-cytokine receptor interaction	IL17A → IL17RA (−0.32)

hsa04310	Wnt signaling pathway	SFRP5 → WNT11 (+0.3)

hsa04520	Adherens junction	WAS → ACTB (−0.39)

hsa04530	Tight junction	PRKCQ → ACTB (−0.34)

hsa04612	Antigen processing and presentation	HLA-E → KIR2DS1 (−0.29)

hsa04620	Toll-like receptor signaling pathway	RIPK1 → TRAF6 (+0.31)

hsa04630	Jak-STAT signaling pathway	STAT6 → SOCS1 (+0.31)

hsa04666	Fc gamma R-mediated phagocytosis	WASF3 → ARPC2 (+0.3)

hsa04725	Cholinergic synapse	GNB5 → PIK3R5 (+0.31)

hsa05012	Parkinson's disease	SLC25A4 → CYCS (+0.28)

hsa05020	Prion diseases	PRNP → BAX (+0.29)

hsa05152	Tuberculosis	MAPK3 → IL23A (+0.31)

hsa05211	Renal cell carcinoma	EGLN3 → EPAS1 (+0.28)

*cor_Dif: average correlation of genes in nonrelapse samples − average correlation of genes in relapse samples.
