# Supplementary material for: A Novel Approach for Discovering Condition-Specific Correlations of Gene Expressions within Biological Pathways by Using Cloud Computing Technology
Source: Biomed Res Int. 2014 Jan 22;2014:763237. doi: 10.1155/2014/763237 (PMC3919110; doi:10.1155/2014/763237)
Supplement: Supplementary file 1 — Figure S1. The differential correlation of gene expression between relapse and nonrelapse samples in pathways in cancer of the KEGG. Table S1: Correlations of gene expressions between nonrelapse and relapse samples in three data sets. Table S2: Gene expression correlations between relapse and nonrelapse samples in Pathways in Cancer. [file 763237.f1.zip › 763237.f1/Table S1.docx]

Table S1. Correlations of gene expressions between nonrelapse and relapse samples in three data sets

|  | Condition  (# of samples) | Number of correlated gene pairs | | Number of differential correlations  of gene pairs (AVG ± 3*SD) |
| --- | --- | --- | --- | --- |
|  |  | Positive (+) Cor. > 0.45 | Negative (-) Cor. < -0.45 |  |
| GSE2034 | Nonrelapse (179)  relapse (107) | 1,857,418  1,595,963 | 279,055  149,044 | 239,400 |
| GSE1456 | Nonrelapse (119)  relapse (40) | 1,856,326 3,630,906 | 224,855 1,331,592 | 229,537 |
| GSE4922 | Nonrelapse (160)  relapse (89) | 1,987,909 2,456,985 | 365,742 507,329 | 279,742 |
