# Supplementary material for: A Novel Approach for Discovering Condition-Specific Correlations of Gene Expressions within Biological Pathways by Using Cloud Computing Technology
Source: Biomed Res Int. 2014 Jan 22;2014:763237. doi: 10.1155/2014/763237 (PMC3919110; doi:10.1155/2014/763237)
Supplement: Supplementary file 1 — Figure S1. The differential correlation of gene expression between relapse and nonrelapse samples in pathways in cancer of the KEGG. Table S1: Correlations of gene expressions between nonrelapse and relapse samples in three data sets. Table S2: Gene expression correlations between relapse and nonrelapse samples in Pathways in Cancer. [file 763237.f1.zip › 763237.f1/Table S1.pdf]

Table S1. Correlations of gene expressions between nonrelapse and relapse samples in three data sets

	Condition (# of samples)	Number of correlated gene pairs		Number of differential correlations of gene pairs (AVG ± 3*SD)
		Positive (+) Cor. > 0.45	Negative (-) Cor. < -0.45	
GSE2034	Nonrelapse (179)	1,857,418	279,055	239,400
	relapse (107)	1,595,963	149,044	
GSE1456	Nonrelapse (119)	1,856,326	224,855	229,537
	relapse (40)	3,630,906	1,331,592	
GSE4922	Nonrelapse (160)	1,987,909	365,742	279,742
	relapse (89)	2,456,985	507,329	
