# Supplementary figures and images for: A Novel Approach for Discovering Condition-Specific Correlations of Gene Expressions within Biological Pathways by Using Cloud Computing Technology
Source: Biomed Res Int. 2014 Jan 22;2014:763237. doi: 10.1155/2014/763237 (PMC3919110; doi:10.1155/2014/763237)

## Slide 1
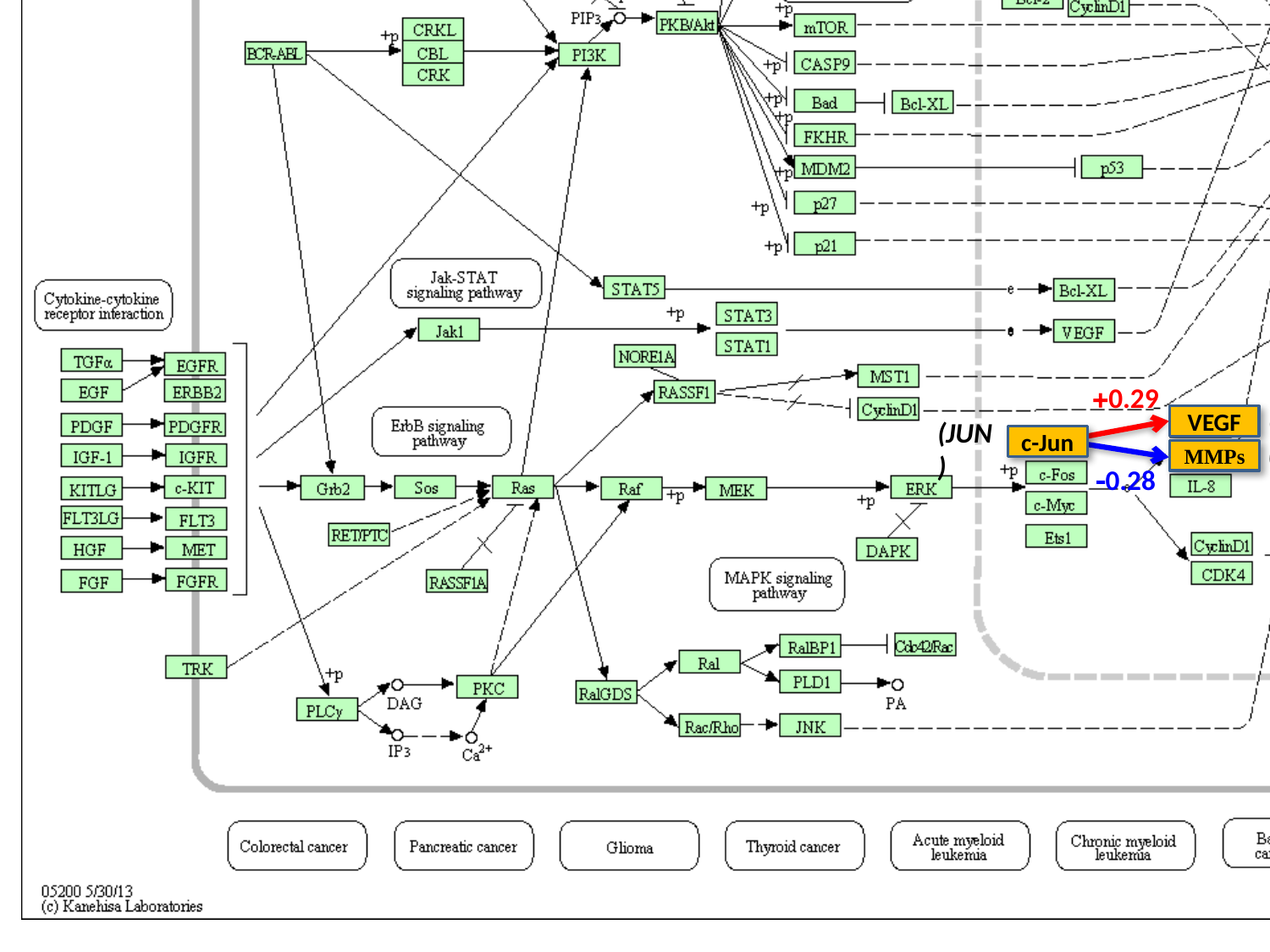

+0.37
COX-2
(PTGS2)
NKkB
(NKFB2)
+0.29
(FIGF)
VEGF
(JUN)
c-Jun
(MMP1)
MMPs
−0.28
C/EBPα
+0.29
(CEBPA)
AML1-ETC
(RUNX1)

Supplement: Supplementary file 1 — Figure S1. The differential correlation of gene expression between relapse and nonrelapse samples in pathways in cancer of the KEGG. Table S1: Correlations of gene expressions between nonrelapse and relapse samples in three data sets. Table S2: Gene expression correlations between relapse and nonrelapse samples in Pathways in Cancer. [file 763237.f1.zip › 763237.f1/Figure S1_The differential gene expression correlations between relapse and nonrelapse samples in pathways in cancer of the KEGG.pptx]
